# Plasma urea cycle metabolite levels and the risk of moyamoya disease

**DOI:** 10.3389/fnins.2023.1163733

**Published:** 2023-07-10

**Authors:** Xiaofan Yu, Peicong Ge, Yuanren Zhai, Wei Liu, Qian Zhang, Xun Ye, Xingju Liu, Rong Wang, Yan Zhang, Jizong Zhao, Dong Zhang

**Affiliations:** ^1^Department of Neurosurgery, Beijing Tiantan Hospital, Capital Medical University, Beijing, China; ^2^China National Clinical Research Center for Neurological Diseases, Beijing, China; ^3^Center of Stroke, Beijing Institute for Brain Disorders, Beijing, China; ^4^Beijing Key Laboratory of Translational Medicine for Cerebrovascular Disease, Beijing, China; ^5^Beijing Translational Engineering Center for 3D Printer in Clinical Neuroscience, Beijing, China; ^6^Savaid Medical School, University of Chinese Academy of Sciences, Beijing, China; ^7^Department of Neurosurgery, Beijing Hospital, Beijing, China

**Keywords:** moyamoya disease, urea cycle, ornithine, arginine, risk factors

## Abstract

**Background and purpose:**

Urea cycle metabolites are expected to be the biomarkers for cerebrovascular diseases. However, the effects of circulating urea cycle metabolites on the risk of MMD and its subcategories remain unclear. The aim of this study was to prospectively investigate the association between plasma urea cycle metabolites and the risk of MMD and its subcategories.

**Methods:**

We measured plasma urea cycle metabolite levels for 360 adult MMD patients and 89 matched healthy controls. Clinical and laboratory characteristics were obtained from the medical record. The study was conducted from July 2020 to December 2021.

**Results:**

After multivariate adjustment, the risk of MMD increased with each increment in ornithine level (per natural log [ornithine] increment: OR, 3.893; 95% CI, 1.366–11.090). The risk of MMD decreased with each increment in arginine level (per natural log [arginine] increment: OR, 0.109; 95% CI, 0.028–0.427), urea level (per natural log [urea] increment: OR, 0.261; 95% CI, 0.072–0.940), and global arginine bioavailability ratio (GABR) level (per natural log [GABR] increment: OR, 0.189; 95% CI, 0.074–0.484). The addition of plasma arginine (integrated discrimination improvement: 1.76%, *p* = 0.021) or GABR (integrated discrimination improvement: 1.76%, *p* = 0.004) to conventional risk factors significantly improved the risk reclassification for MMD.

**Conclusion:**

Plasma ornithine levels are positively associated with the risk of MMD. By contrast, the levels of arginine, urea, and GABR are inversely related to the risk of MMD. Plasma urea cycle metabolites might be potential biomarkers for the risk of MMD.

## Introduction

A rare cerebrovascular condition known as moyamoya disease (MMD) is characterized by the progression of intracranial carotid artery stenosis and an abnormal vascular network in the brain (Suzuki and Takaku, 1969; Scott and Smith, 2009).

The pathogenesis of MMD is presently not well understood. The onset and progression of MMD may be influenced by immune, inflammatory, and genetic causes (Fujimura et al., 2014; Bang et al., 2016). A significant correlation between RNF213 and MMD has been identified (Fujimura et al., 2014; Kim et al., 2016). A polymorphism in RNF213 was identified in 95% of familial patients with MMD and 79% of sporadic cases (Kim et al., 2016). The prevalence of this variant differed widely between MMD patients in Japan (90%) and China (23%) (Miyatake et al., 2012; Zhang et al., 2017). As a result, in addition to hereditary factors, other variables are also important in the onset of MMD in Chinese patients. Previous research has discovered that the risk of MMD is related to a number of conventionally modifiable risk variables, including albumin (ALB), homocysteine (Hcy), body mass index (BMI), and high-density lipoprotein cholesterol (HDL-C) (Ge et al., 2020). Nonetheless, the conventional risk variables are unable to fully account for the MMD risk. It is urgently necessary to identify new risk variables, especially modifiable risk variables.

The relationship between urea cycle metabolites and cardio-cerebrovascular diseases has been extensively studied (Martínez-González et al., 2015; Ahmad et al., 2016; Sun et al., 2019). Ornithine is an essential building block for the production of proline, polyamines, and citrulline and is crucial for the control of a number of metabolic activities (Sivashanmugam et al., 2017). Arginine is an amino acid associated with many biological processes, including protein synthesis, immune reaction, and the creation of nitric oxide. It has been reported that arginine may be a protective factor in ischemic cerebrovascular diseases (Ahmad et al., 2016; Grosse et al., 2020). Furthermore, the concept of “global arginine bioavailability ratio (GABR)” (defined as arginine/[ornithine + citrulline]), which was proposed in 2009, could be more predictive of the occurrence and development of cardio-cerebrovascular diseases (Tang et al., 2009). Although urea is the end product of the urea cycle, it has been associated with the development of stroke (You et al., 2018; Peng et al., 2021). Therefore, urea cycle metabolites are expected to be the biomarkers for cerebrovascular diseases. However, the effects of circulating urea cycle metabolites on the risk of MMD remain unclear. The purpose of this prospective study was to investigate the relationship between the concentrations of urea cycle metabolites and the risk of MMD and its subcategories.

## Methods

Upon reasonable request, the corresponding authors will provide the data supporting the findings of this manuscript. From July 1, 2020, to December 31, 2021, we prospectively collected adult MMD patients (18 years ≤ age ≤ 60 years) at the Department of Neurosurgery, Beijing Tiantan Hospital, Capital Medical University. Each participant signed informed consent. The Ethics Committee of Beijing Tiantan Hospital, Capital Medical University, accepted the study’s procedure (Ethical inspection No. 2021YFC2500502). The registration number of this study is ChiCTR2000031412.

### Study participants

According to the Japanese guidelines released in 2012, digital subtraction angiography (DSA) was used to diagnose all patients with MMD (Research Committee on the Pathology and Treatment of Spontaneous Occlusion of the Circle of Willis and Health Labour Sciences Research Grant for Research on Measures for Intractable Diseases, 2012). A total of 500 patients with MMD-type cerebrovascular disease (including 418 adult patients) from across the nation received care at our facility from July 1, 2020, to December 31, 2021. Among the adult patients, 11 patients older than 60 years, 13 patients with moyamoya syndrome, and 34 patients without urea cycle metabolite data were excluded. Moyamoya syndrome refers to patients with MMD-type cerebrovascular disease accompanied by other basic diseases, such as arteriosclerosis, autoimmune disease, meningitis, and Down syndrome (Scott and Smith, 2009). Finally, 360 adult patients with comprehensive urea cycle metabolite data were included in this study (Supplementary Figure I). Among the 360 adult patients, 259 patients had ischemic-type MMD and 101 patients had hemorrhagic-type MMD. Age-matched healthy individuals who underwent regular checkups were recruited for the control group. The individuals in the control group did not suffer from heart disease or MMD, according to interviews with them and their families.

### Data collection

After a 15-min break spent seated, the participants’ right arms were examined for systolic blood pressure (SBP) and diastolic blood pressure (DBP) using a conventional mercury manometer. Additionally, electrocardiography heart rate (HR) data were captured. Weight (kg)/height (m^2^) was used to determine the BMI. Venous blood samples were collected from the participants after 12 h of fasting. White blood cells (WBC), lymphocytes (LY), platelets (PLT), glucose (Glu), ALB, creatinine (Cr), uric acid (UA), triglyceride (TG), total cholesterol (TC), HDL-C, low-density lipoprotein cholesterol (LDL-C), apolipoprotein A (apoA), apolipoprotein B (apoB), and Hcy were all measured in fasting blood. The RNF213 p.R4810K variant was detected, but the p.A4399T variant was not detected. The following primers were used: RNF213-4810F (rs112735431), 5′-GCCCTCCATTTCTAGCACAC-3′, and RNF21-4810R, 5′-AGCTGTGGCGAAAGCTTCTA-3′.

Data on MMD patients’ age, sex, and comorbidities, such as hypertension, coronary artery disease, smoking, drinking, diabetes mellitus, thyroid disorders, and initial clinical features (ischemia and hemorrhage), were gathered at the time of admission. Smokers were participants who smoked at least one cigarette per day for at least 1 year. Drinkers were individuals who drank at least 125 mL of wine per day for at least 1 year (D' Avanzo et al., 1996). Two neurosurgeons used DSA to evaluate the Suzuki stage blindly.

### Quantification of urea cycle metabolites

The plasma levels of urea cycle metabolites (ornithine, citrulline, arginine, aspartic acid, and urea) were quantified by ultra-performance liquid chromatography tandem mass spectrometry (Sciex QTRAP 6500 LC–MS/MS). An aliquot of 50 μL of the serum sample was transferred to a centrifuge tube, mixed with 250 μL of 20% acetonitrile/methanol, vortexed for 3 min, and centrifuged at 12,000 rpm for 10 min at 4°C. Then, 250 μL of the supernatant was transferred to a new centrifuge tube and placed in a freezer at −20°C for 30 min. Next, the supernatant was centrifuged at 12,000 rpm for 10 min at 4°C. After centrifugation, 180 μL of the supernatant was added to a protein precipitation plate for further LC–MS analysis.

### Statistical analysis

Baseline characteristics are presented as medians (25th–75th percentile) for continuous variables and proportions for categorical variables. Chi-square and Mann–Whitney U tests were used for categorical and continuous variables in baseline features, respectively.

The participants were divided into four categories based on the quartile of change in ornithine, arginine, urea, or GABR index. *P* for trend was calculated using the quartile of change in ornithine, arginine, urea, or GABR index as the ordinal variable. The Cochran–Armitage test was used to assess trends in categorical variables, and one-way ANOVA was performed to analyze continuous variables.

Odds ratios (ORs) of the risk of MMD (MMD overall, ischemic MMD, and hemorrhagic MMD) to plasma levels of ornithine, arginine, urea, or GABR (continuous and categorical variables) were assessed by a conditional logistic regression model, without and with modification for age, gender, HR, SBP, DBP, BMI, WBC, LY, PLT, Glu, Cr, UA, ALB, TG, TC, HDL-C, LDL-C, apoA, apoB, and Hcy.

Two statistical indices were used to measure the improvement in model performance resulting from the addition of new markers: net reclassification improvement and integrated discrimination improvement (Pencina et al., 2008). We created a conventional model (only including risk factors in model 2) and four novel models (including risk factors in model 2 and plasma ornithine, arginine, urea, or GABR) by using logistic regression. We evaluated net reclassification improvement and integrated discrimination improvement by comparing these models to determine whether adding plasma ornithine, arginine, urea, or GABR to risk factors might enhance the prediction ability for the risk of MMD.

All statistical analysis were carried out using IBM SPSS Statistics (version 22.0; IBM Corp.) and R software (version 4.2.0).^1^ *p* < 0.05 was considered statistically significant, and all tests were two-tailed.

## Results

### Study participants and baseline features

**Figure 1:**
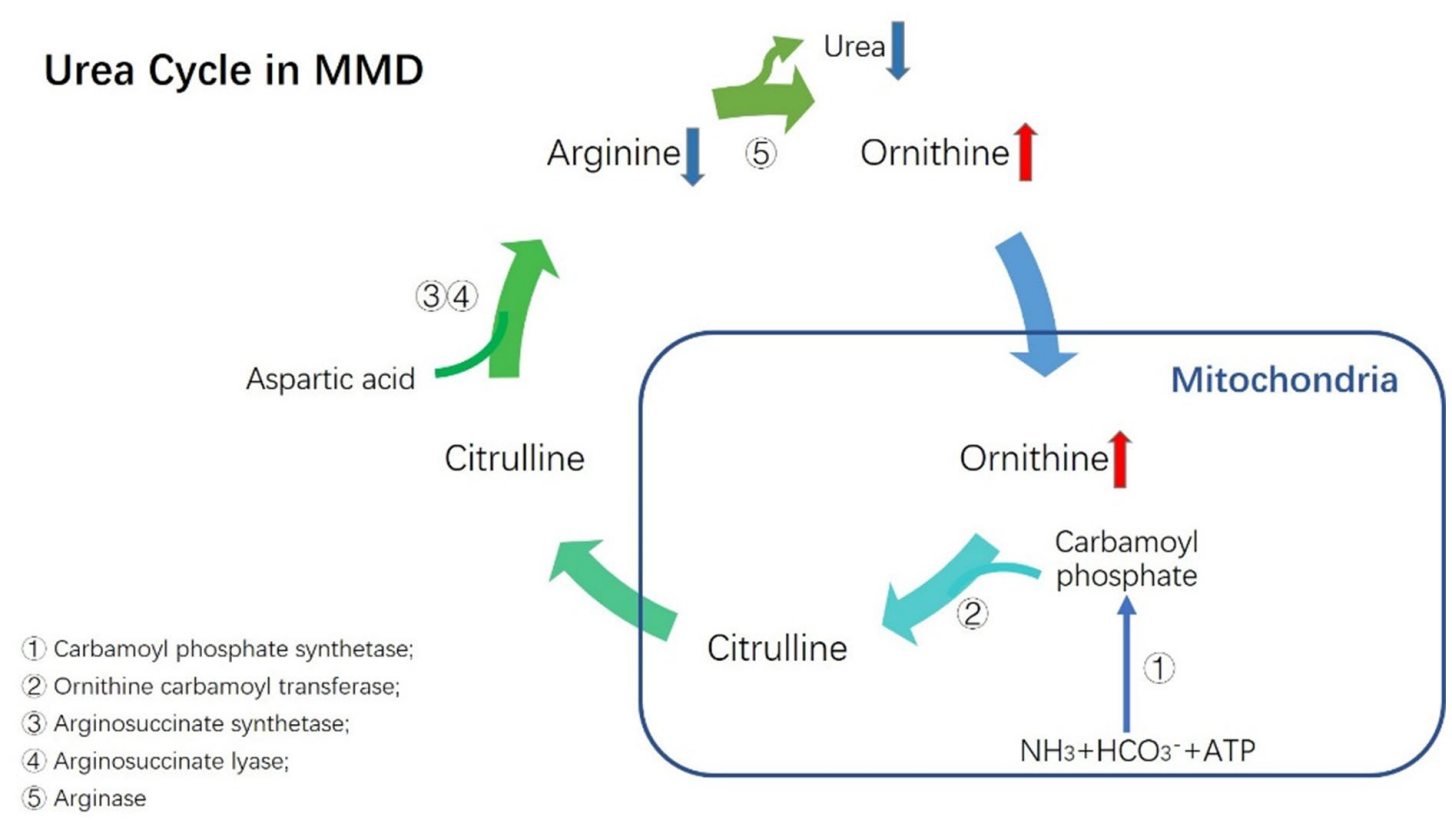
Urea cycle in MMD.

The study was conducted from July 2020 to December 2021. In this study, we analyzed 360 MMD patients (259 patients with ischemic MMD and 101 patients with hemorrhagic MMD) and 89 age-matched controls with comprehensive urea cycle metabolite examinations (Supplementary Figure I). A total of 187 (41.65%) males and 262 (58.35%) females were enrolled, and the median age was 43.00 years (IQR, 33.00–50.00 years). The clinical and laboratory features of MMD and the controls are shown in Table 1. MMD patients had more risk variables for stroke compared with the healthy controls. MMD and its subcategories had higher levels of SBP, WBC count, TG, Hcy, and ornithine, and a higher prevalence of hypertension, hyperlipidemia, thyroid disease, smoking, and drinking (*p* < 0.05 for all). Moreover, patients with MMD had lower levels of HDL-C, apoA, urea, and GABR (*p* < 0.05 for all) (Figure 1). Patients with ischemic MMD had a lower level of arginine (*p* < 0.05). Patients with ischemic or hemorrhagic MMD had a higher prevalence of the RNF213 p.R4810K variant (*p* < 0.05 for all).

**Table 1:** Clinical and laboratory characteristics in MMD patients and healthy controls.

Variables	Controls (*n* = 89)	MMD (*n* = 360)	*p*	Ischemic-type MMD (*n* = 259)	*p*	Hemorrhagic-type MMD (*n* = 101)	*p*
Age, y	39.00 (31.00–50.00)	43.00 (34.00–49.00)	0.209	43.00 (34.00–49.00)	0.251	43.00 (33.50–50.00)	0.248
Men (%)	37 (41.57)	150 (41.67)	0.987	116 (44.79)	0.598	34 (33.66)	0.261
RNF213 p.R4810K	0/89	60/255	**0.000**	44/185	**0.000**	16/70	**0.006**
Heart rate, bpm	78.00 (70.00–85.00)	78.00 (75.00–80.00)	0.114	78.00 (75.00–80.00)	0.239	79.00 (76.00–82.00)	**0.043**
SBP, mmHg	124.00 (115.50–130.00)	132.00 (124.00–140.00)	**0.000**	134.00 (125.00–140.00)	**0.000**	130.00 (121.50–138.00)	**0.001**
DBP, mmHg	78.00 (74.00–82.00)	81.00 (76.00–89.00)	**0.001**	82.00 (77.00–90.00)	**0.000**	80.00 (74.00–88.00)	0.090
BMI, kg/m^2^	23.51 (21.41–26.47)	25.00 (22.49–27.78)	**0.007**	25.35 (23.03–28.13)	**0.000**	24.03 (21.92–26.20)	0.844
Medical history (%)							
Hypertension	0 (0)	131 (36.89)	**0.000**	102 (39.38)	**0.000**	29 (28.71)	**0.000**
Diabetes	0 (0)	59 (16.39)	**0.000**	55 (21.24)	**0.000**	4 (3.96)	0.058
Coronary artery disease	0 (0)	7 (1.94)	**0.185**	5 (1.93)	0.187	2 (1.98)	0.182
Hyperlipidemia	0 (0)	54 (15.00)	**0.000**	45 (17.37)	**0.000**	9 (8.91)	**0.004**
Thyroid disease	0 (0)	21 (5.83)	**0.020**	16 (6.18)	**0.016**	5 (4.95)	**0.033**
Smoking	2 (2.25)	71 (19.72)	**0.000**	54 (20.85)	**0.000**	17 (16.83)	**0.001**
Drinking	0 (0)	42 (11.67)	**0.001**	34 (13.13)	**0.000**	8 (7.92)	**0.007**
Laboratory results, median (IQR)							
WBC count, 10^9^/L	6.03 (5.01–6.89)	6.81 (5.64–8.12)	**0.000**	6.97 (5.77–8.19)	**0.000**	6.43 (5.40–7.86)	**0.018**
Lymphocyte count, 10^9^/L	1.91 (1.52–2.23)	1.92 (1.54–2.41)	0.247	2.03 (1.60–2.48)	**0.023**	1.72 (1.38–2.22)	0.120
Platelet count, 10^9^/L	233.00 (201.50–288.50)	248.00 (209.50–284.75)	0.271	250.00 (215.00–288.00)	0.149	244.00 (204.00–279.50)	0.944
Glucose, mmol/L	5.04 (4.75–5.37)	5.11 (4.71–5.77)	0.229	5.16 (4.77–5.91)	**0.016**	4.91 (4.56–5.23)	0.084
Creatinine, μmol/L	57.70 (49.35–68.55)	54.75 (46.33–66.98)	0.151	55.70 (46.80–67.80)	0.284	53.10 (45.20–66.95)	0.065
Uric acid, μmol/L	310.60 (250.80–354.25)	305.60 (254.33–369.78)	0.743	312.00 (257.50–378.90)	0.340	292.90 (241.65–354.95)	0.265
Albumin, g/L	44.90 (43.50–46.65)	45.50 (43.40–47.38)	0.330	45.50 (43.40–47.40)	0.288	45.30 (43.45–47.10)	0.600
Triglyceride, mmol/L	0.87 (0.65–1.26)	1.20 (0.82–1.64)	**0.000**	1.22 (0.84–1.64)	**0.000**	1.13 (0.79–1.64)	**0.008**
Total cholesterol, mmol/L	4.62 (4.08–5.06)	4.23 (3.55–4.83)	**0.000**	4.09 (3.39–4.71)	**0.000**	4.35 (3.90–5.03)	0.194
HDL-C, mmol/L	1.53 (1.33–1.74)	1.30 (1.12–1.49)	**0.000**	1.27 (1.09–1.47)	**0.000**	1.34 (1.17–1.52)	**0.001**
LDL-C, mmol/L	2.69 (2.26–3.13)	2.40 (1.84–2.98)	**0.001**	2.23 (1.76–2.90)	**0.000**	2.55 (2.19–3.26)	0.762
apoA, g/L	1.39 (1.26–1.54)	1.29 (1.15–1.45)	**0.000**	1.29 (1.13–1.44)	**0.000**	1.30 (1.17–1.46)	**0.005**
apoB, g/L	0.77 (0.69–0.95)	0.82 (0.70–0.97)	0.329	0.82 (0.68–0.95)	0.706	0.82 (0.71–1.02)	**0.044**
Homocysteine	10.62 (8.67–12.63)	12.00 (9.30–15.08)	**0.001**	12.00 (9.27–15.61)	**0.001**	11.90 (9.38–14.35)	**0.006**
Ornithine, ng/mL	32.05 (24.02–38.96)	36.12 (29.45–43.41)	**0.000**	36.55 (30.20–43.27)	**0.000**	34.19 (28.71–43.89)	**0.033**
Citrulline, ng/mL	20.55 (16.76–24.83)	20.64 (16.72–24.84)	0.999	20.59 (16.94–24.54)	0.897	21.10 (16.37–25.98)	0.776
Arginine, ng/mL	73.29 (60.77–83.72)	65.61 (56.85–75.42)	**0.002**	64.70 (56.20–74.58)	**0.001**	68.01 (57.38–79.02)	0.055
Aspartic acid, ng/mL	57.67 (47.29–72.07)	55.47 (47.27–66.92)	0.293	55.61 (47.49–65.95)	0.266	54.90 (46.21–71.19)	0.523
Urea, mmol/L	5.97 (5.17–7.45)	5.60 (4.82–6.45)	**0.002**	5.57 (4.84–6.38)	**0.001**	5.67 (4.80–6.54)	**0.026**
GABR, median (IQR)	1.37 (1.07–1.80)	1.18 (0.96–1.44)	**0.000**	1.17 (0.94–1.39)	**0.000**	1.23 (1.02–1.50)	**0.020**

SBP, systolic blood pressure; DBP, diastolic blood pressure; BMI, body mass index; WBC, white blood cells; HDL-C, high-density lipoprotein cholesterol; LDL-C, low-density lipoprotein cholesterol; apoA, apolipoprotein A; apoB, apolipoprotein B; MMD, moyamoya disease; GABR, global arginine bioavailability ratio. The bold values mean *p*-value < 0.05.

### Urea cycle metabolites and the risk of MMD

The correlations of plasma ornithine, arginine, urea, and GABR levels with the risk of MMD are presented in Tables 2–5. After adjustment for model 2, the risk of MMD increased with each increment in ornithine level (per natural log [ornithine] increment: OR, 3.893; 95% CI, 1.366–11.090). When plasma ornithine levels were divided into quartiles, those in the highest quartile had a noticeably higher risk of MMD than those in the lowest quartile (Q4 versus Q1: OR, 3.626; 95% CI, 1.334–9.855). Conversely, the risk of MMD decreased with each increment in arginine level (per natural log [arginine] increment: OR, 0.109; 95% CI, 0.028–0.427), urea level (per natural log [urea] increment: OR, 0.261; 95% CI, 0.072–0.940), and GABR level (per natural log [GABR] increment: OR, 0.189; 95% CI, 0.074–0.484) after adjustment for the risk variables in model 2. When plasma levels of arginine, urea, and GABR were divided into quartiles, those in the highest quartile had a noticeably lower risk of MMD than those in the lowest quartile (arginine, Q4 versus Q1: OR, 0.273; 95% CI, 0.114–0.652; urea, Q4 versus Q1: OR, 0.318; 95% CI, 0.136–0.745; GABR, Q4 versus Q1: OR, 0.264; 95% CI, 0.108–0.649). We can see the association of urea cycle metabolites and conventional risk factors in the Supplementary Materials (Supplementary Tables I–IV). Participants with higher ornithine concentrations tended to be male and had higher BMI, Glu, Cr, UA, apoB, and Hcy levels, lower PLT, HDL-C, and apoA levels, and a higher prevalence of hypertension, diabetes, coronary artery disease, smoking, and drinking. Participants with higher arginine concentrations tended to be male and had higher BMI, PLT, and LDL-C levels. Participants with higher urea concentrations tended to be male and had higher Cr and UA levels and a higher prevalence of coronary artery disease. Participants with higher GABR levels tended to be female and had higher PLT and HDL-C levels, lower Cr, UA, and Hcy levels, and a lower prevalence of smoking.

**Table 2:** The association between ornithine and the risk of MMD.

Ornithine, ng/mL	No. of events (%)	Crude		Model 1^*^		Model 2^†^	
		OR (95% CI)	*P* Value	OR (95% CI)	*P* Value	OR (95% CI)	*P* Value
MMD overall							
Continuous	360 (80.18)	1.035 (1.011–1.060)	**0.004**	1.031 (1.004–1.060)	**0.027**	1.032 (1.000–1.064)	**0.049**
Natural log transformed	360 (80.18)	3.858 (1.839–8.097)	**0.000**	3.739 (1.527–9.155)	**0.004**	3.893 (1.366–11.090)	**0.011**
Quartiles							
Q1 (<28.61)	78 (69.64)	1.0 (Ref)		1.0 (Ref)		1.0 (Ref)	
Q2 (28.61 – < 35.61)	91 (80.53)	1.803 (0.974–3.337)	0.061	1.678 (0.853–3.302)	0.134	1.395 (0.633–3.072)	0.409
Q3 (35.61 – < 42.84)	92 (82.14)	2.005 (1.069–3.762)	**0.030**	2.055 (1.014–4.161)	**0.046**	1.684 (0.730–3.884)	0.222
Q4 (≥42.84)	99 (88.39)	3.320 (1.641–6.716)	**0.001**	3.266 (1.422–7.500)	**0.005**	3.626 (1.334–9.855)	**0.012**
*P* for trend		0.001		0.004		0.014	
Ischemic MMD							
Continuous	259 (57.68)	1.038 (1.013–1.063)	**0.003**	1.034 (1.004–1.066)	**0.027**	1.037 (1.000–1.074)	**0.047**
Natural log transformed	259 (57.68)	4.185 (1.924–9.101)	**0.000**	4.277 (1.584–11.546)	**0.004**	4.846 (1.418–16.564)	**0.012**
Quartiles							
Q1 (<28.61)	54 (61.36)	1.0 (Ref)		1.0 (Ref)		1.0 (Ref)	
Q2 (28.61 – < 35.61)	60 (73.17)	1.717 (0.896–3.290)	0.103	1.637 (0.775–3.456)	0.196	1.345 (0.523–3.461)	0.539
Q3 (35.61 – < 42.84)	73 (78.49)	2.298 (1.194–4.424)	**0.013**	2.203 (1.031–4.708)	**0.042**	1.795 (0.695–4.634)	0.227
Q4 (≥42.84)	72 (84.71)	3.487 (1.680–7.237)	**0.001**	3.809 (1.521–9.543)	**0.004**	5.091 (1.563–16.586)	**0.007**
*P* for trend		0.000		0.003		0.008	
Hemorrhagic MMD							
Continuous	101 (22.49)	1.023 (0.998–1.049)	0.069	1.025 (0.996–1.054)	0.093	1.033 (0.998–1.068)	0.067
Natural log transformed	101 (22.49)	2.756 (1.145–6.633)	**0.024**	3.114 (1.121–8.647)	**0.029**	4.433 (1.248–15.747)	**0.021**
Quartiles							
Q1 (<28.61)	24 (41.38)	1.0 (Ref)		1.0 (Ref)		1.0 (Ref)	
Q2 (28.61 – < 35.61)	31 (58.49)	1.996 (0.937–4.252)	0.073	1.949 (0.863–4.399)	0.108	1.979 (0.745–5.254)	0.171
Q3 (35.61 – < 42.84)	19 (48.72)	1.346 (0.595–3.046)	0.476	1.567 (0.648–3.792)	0.319	2.011 (0.666–6.066)	0.215
Q4 (≥42.84)	27 (67.50)	2.942 (1.266–6.837)	**0.012**	3.282 (1.241–8.684)	**0.017**	4.502 (1.275–15.893)	**0.019**
*P* for trend		0.032		0.034		0.028	

MMD, moyamoya disease; OR, odds ratio.

^*^Model 1 was adjusted for age, gender, heart rate, SBP, DBP, and BMI.

^†^Model 2 was adjusted for all the variables in model 1 plus WBC count, lymphocyte count, platelet count, glucose, creatinine, uric acid, albumin, triglyceride, total cholesterol, HDL-C, LDL-C, apoA, apoB, and homocysteine.

The bold values mean *p*-value < 0.05.

**Table 3:** The association between arginine and the risk of MMD.

Arginine, ng/mL	No. of events (%)	Crude		Model 1^*^		Model 2^†^	
		OR (95% CI)	*P* Value	OR (95% CI)	*P* Value	OR (95% CI)	*P* Value
MMD overall							
Continuous	360 (80.18)	0.985 (0.972–0.998)	**0.024**	0.979 (0.965–0.993)	**0.003**	0.974 (0.957–0.992)	**0.005**
Natural log transformed	360 (80.18)	0.267 (0.099–0.721)	**0.009**	0.160 (0.053–0.477)	**0.001**	0.109 (0.028–0.427)	**0.001**
Quartiles							
Q1 (<57.38)	95 (84.82)	1.0 (Ref)		1.0 (Ref)		1.0 (Ref)	
Q2 (57.38 – < 66.41)	93 (83.04)	0.876 (0.429–1.789)	0.716	0.786 (0.369–1.675)	0.533	0.682 (0.280–1.664)	0.401
Q3 (66.41 – < 78.41)	93 (82.30)	0.832 (0.410–1.687)	0.610	0.726 (0.346–1.527)	0.399	0.873 (0.368–2.073)	0.759
Q4 (≥78.41)	79 (70.54)	0.428 (0.222–0.826)	**0.011**	0.319 (0.157–0.647)	**0.002**	0.273 (0.114–0.652)	**0.003**
*P* for trend		0.010		0.002		0.009	
Ischemic MMD							
Continuous	259 (57.68)	0.982 (0.968–0.996)	**0.012**	0.975 (0.959–0.991)	**0.002**	0.965 (0.944–0.986)	**0.001**
Natural log transformed	259 (57.68)	0.219 (0.076–0.631)	**0.005**	0.128 (0.039–0.422)	**0.001**	0.059 (0.012–0.296)	**0.001**
Quartiles							
Q1 (<57.38)	70 (80.46)	1.0 (Ref)		1.0 (Ref)		1.0 (Ref)	
Q2 (57.38 – < 66.41)	71 (78.89)	0.908 (0.436–1.889)	0.795	0.820 (0.368–1.827)	0.628	0.821 (0.303–2.223)	0.698
Q3 (66.41 – < 78.41)	65 (76.47)	0.789 (0.381–1.637)	0.525	0.618 (0.281–1.359)	0.231	0.841 (0.312–2.269)	0.733
Q4 (≥78.41)	53 (61.63)	0.390 (0.197–0.774)	**0.007**	0.294 (0.137–0.629)	**0.002**	0.237 (0.087–0.647)	**0.005**
*P* for trend		0.005		0.001		0.008	
Hemorrhagic MMD							
Continuous	101 (22.49)	0.989 (0.972–1.007)	0.221	0.983 (0.965–1.002)	0.081	0.980 (0.957–1.003)	0.086
Natural log transformed	101 (22.49)	0.372 (0.103–1.343)	0.131	0.239 (0.060–0.954)	**0.043**	0.162 (0.028–0.936)	**0.042**
Quartiles							
Q1 (<57.38)	25 (59.52)	1.0 (Ref)		1.0 (Ref)		1.0 (Ref)	
Q2 (57.38 – < 66.41)	22 (53.66)	0.787 (0.330–1.879)	0.590	0.883 (0.350–2.227)	0.792	0.613 (0.189–1.990)	0.415
Q3 (66.41 – < 78.41)	28 (58.33)	0.952 (0.410–2.210)	0.909	0.965 (0.399–2.334)	0.937	1.147 (0.387–3.403)	0.804
Q4 (≥78.41)	26 (44.07)	0.536 (0.240–1.195)	0.127	0.444 (0.188–1.045)	0.063	0.348 (0.114–1.059)	0.063
*P* for trend		0.164		0.075		0.136	

MMD, moyamoya disease; OR, odds ratio.

^*^Model 1 was adjusted for age, gender, heart rate, SBP, DBP, and BMI.

^†^Model 2 was adjusted for all the variables in model 1 plus WBC count, lymphocyte count, platelet count, glucose, creatinine, uric acid, albumin, triglyceride, total cholesterol, HDL-C, LDL-C, apoA, apoB, and homocysteine.

The bold values mean *p*-value < 0.05.

**Table 4:** The association between urea and the risk of MMD.

Urea, ng/mL	No. of events (%)	Crude		Model 1^*^		Model 2^†^	
		OR (95% CI)	*P* Value	OR (95% CI)	*P* Value	OR (95% CI)	*P* Value
MMD overall							
Continuous	360 (80.18)	0.764 (0.646–0.903)	**0.002**	0.731 (0.607–0.879)	**0.001**	0.779 (0.623–0.974)	**0.029**
Natural log transformed	360 (80.18)	0.218 (0.080–0.592)	**0.003**	0.173 (0.059–0.511)	**0.002**	0.261 (0.072–0.940)	**0.040**
Quartiles							
Q1 (<4.87)	96 (85.71)	1.0 (Ref)		1.0 (Ref)		1.0 (Ref)	
Q2 (4.87 – < 5.69)	93 (83.04)	0.816 (0.396–1.682)	0.581	0.632 (0.293–1.362)	0.241	0.597 (0.238–1.494)	0.270
Q3 (5.69 – < 6.59)	96 (84.96)	0.941 (0.450–1.971)	0.872	0.899 (0.411–1.968)	0.790	1.068 (0.442–2.581)	0.884
Q4 (≥6.59)	75 (66.96)	0.338 (0.175–0.653)	**0.001**	0.281 (0.136–0.580)	**0.001**	0.318 (0.136–0.745)	**0.008**
*P* for trend		0.001		0.001		0.024	
Ischemic MMD							
Continuous	259 (57.68)	0.742 (0.619–0.888)	**0.001**	0.688 (0.558–0.847)	**0.000**	0.751 (0.575–0.981)	**0.036**
Natural log transformed	259 (57.68)	0.193 (0.067–0.557)	**0.002**	0.133 (0.039–0.446)	**0.001**	0.248 (0.056–1.102)	0.067
Quartiles							
Q1 (<4.87)	69 (81.18)	1.0 (Ref)		1.0 (Ref)		1.0 (Ref)	
Q2 (4.87 – < 5.69)	69 (78.41)	0.842 (0.400–1.772)	0.651	0.591 (0.261–1.340)	0.208	0.469 (0.161–1.364)	0.165
Q3 (5.69 – < 6.59)	68 (80.00)	0.928 (0.434–1.984)	0.846	0.876 (0.378–2.028)	0.757	1.206 (0.445–3.270)	0.712
Q4 (≥6.59)	53 (58.89)	0.332 (0.167–0.660)	**0.002**	0.236 (0.106–0.524)	**0.000**	0.288 (0.104–0.799)	**0.017**
*P* for trend		0.002		0.001		0.076	
Hemorrhagic MMD							
Continuous	101 (22.49)	0.807 (0.661–0.986)	**0.036**	0.789 (0.634–0.982)	**0.034**	0.785 (0.598–1.032)	0.083
Natural log transformed	101 (22.49)	0.294 (0.095–0.905)	**0.033**	0.251 (0.074–0.850)	**0.026**	0.231 (0.050–1.071)	0.061
Quartiles							
Q1 (<4.87)	27 (62.79)	1.0 (Ref)		1.0 (Ref)		1.0 (Ref)	
Q2 (4.87 – < 5.69)	24 (55.81)	0.749 (0.316–1.774)	0.511	0.721 (0.289–1.800)	0.484	0.720 (0.226–2.294)	0.578
Q3 (5.69 – < 6.59)	28 (62.22)	0.976 (0.412–2.314)	0.956	0.942 (0.381–2.329)	0.896	0.790 (0.269–2.325)	0.669
Q4 (≥6.59)	22 (37.29)	0.352 (0.156–0.794)	**0.012**	0.338 (0.142–0.806)	**0.014**	0.310 (0.109–0.879)	**0.028**
*P* for trend		0.018		0.021		0.031	

MMD, moyamoya disease; OR, odds ratio.

^*^Model 1 was adjusted for age, gender, heart rate, SBP, DBP, and BMI.

^†^Model 2 was adjusted for all the variables in model 1 plus WBC count, lymphocyte count, platelet count, glucose, creatinine, uric acid, albumin, triglyceride, total cholesterol, HDL-C, LDL-C, apoA, apoB, and homocysteine.

The bold values mean *p*-value < 0.05.

**Table 5:** The association between GABR and the risk of MMD.

Urea, ng/mL	No. of events (%)	Crude		Model 1^*^		Model 2^†^	
		OR (95% CI)	*P* value	OR (95% CI)	*P* value	OR (95% CI)	*P* Value
MMD overall							
Continuous	360 (80.18)	0.357 (0.218–0.585)	**0.000**	0.326 (0.188–0.566)	**0.000**	0.312 (0.164–0.594)	**0.000**
Natural log transformed	360 (80.18)	0.232 (0.111–0.486)	**0.000**	0.207 (0.091–0.468)	**0.000**	0.189 (0.074–0.484)	**0.001**
Quartiles							
Q1 (<0.98)	98 (86.73)	1.0 (Ref)		1.0 (Ref)		1.0 (Ref)	
Q2 (0.98 – < 1.20)	95 (84.82)	0.855 (0.404–1.810)	0.683	0.751 (0.341–1.653)	0.477	0.627 (0.249–1.581)	0.323
Q3 (1.20 – < 1.48)	90 (80.36)	0.626 (0.306–1.281)	0.200	0.596 (0.279–1.277)	0.183	0.501 (0.205–1.224)	0.130
Q4 (≥1.48)	77 (68.75)	0.337 (0.172–0.661)	**0.002**	0.314 (0.150–0.658)	**0.002**	0.264 (0.108–0.649)	**0.004**
*P* for trend		0.001		0.001		0.003	
Ischemic MMD							
Continuous	259 (57.68)	0.353 (0.211–0.590)	**0.000**	0.303 (0.167–0.551)	**0.000**	0.264 (0.127–0.549)	**0.000**
Natural log transformed	259 (57.68)	0.220 (0.103–0.468)	**0.000**	0.181 (0.075–0.434)	**0.000**	0.145 (0.050–0.422)	**0.000**
Quartiles							
Q1 (<0.98)	76 (83.52)	1.0 (Ref)		1.0 (Ref)		1.0 (Ref)	
Q2 (0.98 – < 1.20)	69 (80.23)	0.801 (0.372–1.725)	0.571	0.654 (0.283–1.513)	0.321	0.546 (0.193–1.549)	0.256
Q3 (1.20 – < 1.48)	65 (74.71)	0.583 (0.280–1.216)	0.150	0.547 (0.245–1.222)	0.141	0.473 (0.174–1.289)	0.143
Q4 (≥1.48)	49 (58.33)	0.276 (0.137–0.558)	**0.000**	0.238 (0.106–0.534)	**0.001**	0.201 (0.072–0.557)	**0.002**
*P* for trend		0.000		0.000		0.002	
Hemorrhagic MMD							
Continuous	101 (22.49)	0.381 (0.194–0.751)	**0.005**	0.333 (0.157–0.708)	**0.004**	0.259 (0.103–0.654)	**0.004**
Natural log transformed	101 (22.49)	0.327 (0.131–0.815)	**0.016**	0.277 (0.101–0.759)	**0.013**	0.180 (0.052–0.627)	**0.007**
Quartiles							
Q1 (<0.98)	22 (59.46)	1.0 (Ref)		1.0 (Ref)		1.0 (Ref)	
Q2 (0.98 – < 1.20)	26 (60.47)	1.043 (0.425–2.557)	0.927	0.927 (0.362–2.379)	0.875	0.748 (0.236–2.371)	0.622
Q3 (1.20 – < 1.48)	25 (53.19)	0.775 (0.324–1.852)	0.566	0.688 (0.272–1.739)	0.429	0.403 (0.129–1.262)	0.119
Q4 (≥1.48)	28 (44.44)	0.545 (0.239–1.242)	0.149	0.482 (0.197–1.176)	0.109	0.322 (0.103–1.008)	0.052
*P* for trend		0.086		0.070		0.030	

MMD, moyamoya disease; OR, odds ratio; GABR, global arginine bioavailability ratio.

^*^ Model 1 was adjusted for age, gender, heart rate, SBP, DBP, and BMI.

^†^Model 2 was adjusted for all the variables in model 1 plus WBC count, lymphocyte count, platelet count, glucose, creatinine, uric acid, albumin, triglyceride, total cholesterol, HDL-C, LDL-C, apoA, apoB, and homocysteine.

The bold values mean *p*-value < 0.05.

The Suzuki stage and RNF213 p.R4810K variant in patients with MMD are shown in Supplementary Table V. We did not detect the p.A4399T variant in the present study. The results suggested that MMD patients with lower plasma levels of citrulline or urea were more likely to have the RNF213 p.R4810K variant. Furthermore, MMD patients with higher levels of GABR tended to have a higher Suzuki stage level and the RNF213 p.R4810K variant (*p* < 0.05 for all). In addition, we further analyzed the association between GABR and the risk of hemorrhagic MMD in the overall MMD cases. After adjusting for all the variables in model 2, a higher trend of hemorrhagic MMD (OR, 2.432; 95% CI, 1.088–5.436) was found in MMD patients in Q4 compared with those in Q1 (Supplementary Tables VI).

### Incremental prognostic value of urea cycle metabolites

We examined whether adding plasma urea cycle metabolites (ornithine, arginine, urea, and GABR) to the conventional model (all risk factors in model 2) could improve its ability to predict the risk of MMD. As shown in Table 6, the addition of plasma arginine (net reclassification improvement: 54.18%, *p* = 0.000; integrated discrimination improvement: 1.76%, *p* = 0.021) or GABR (net reclassification improvement: 27.72%, *p* = 0.018; integrated discrimination improvement: 1.76%, *p* = 0.004) to conventional risk factors significantly improved the risk reclassification for overall MMD. The addition of plasma arginine (net reclassification improvement: 59.82%, *p* = 0.000; integrated discrimination improvement: 2.92%, *p* = 0.004) or GABR (net reclassification improvement: 38.58%, *p* = 0.001; integrated discrimination improvement: 3.80%, *p* = 0.003) to conventional risk factors also significantly improved the risk reclassification for ischemic MMD. Furthermore, the addition of GABR (net reclassification improvement: 33.80%, *p* = 0.017; integrated discrimination improvement: 3.95%, *p* = 0.005) to conventional risk factors significantly improved the risk reclassification for hemorrhagic MMD.

**Table 6:** Performance of models with plasma urea cycle metabolites at predicting the risk of MMD and its subtypes.

	Continuous NRI, %		IDI, %	
	Estimate (95% CI)	*P* Value	Estimate (95% CI)	*P* Value
MMD overall				
Conventional model	1.0 (Ref)		1.0 (Ref)	
Conventional model + ornithine	28.60 (5.92–51.27)	**0.013**	1.33 (−0.06–2.71)	0.060
Conventional model + arginine	54.18 (31.79–76.56)	**0.000**	1.76 (0.27–3.26)	**0.021**
Conventional model + urea	34.58 (11.64–57.53)	**0.003**	1.02 (−0.27–2.31)	0.121
Conventional model + GABR	27.72 (4.8–50.63)	**0.018**	3.42 (1.12–5.72)	**0.004**
Ischemic MMD				
Conventional model	1.0 (Ref)		1.0 (Ref)	
Conventional model + ornithine	15.70 (−8.13–39.52)	0.197	1.6 (0.02–3.19)	**0.047**
Conventional model + arginine	59.82 (36.72–82.92)	**0.000**	2.92 (0.96–4.88)	**0.004**
Conventional model + urea	35.07 (11.33–58.81)	**0.004**	1.19 (−0.32–2.7)	0.122
Conventional model + GABR	38.58 (14.95–62.20)	**0.001**	3.80 (1.29–6.32)	**0.003**
Hemorrhagic MMD				
Conventional model	1.0 (Ref)		1.0 (Ref)	
Conventional model + ornithine	33.95 (5.88–62.02)	**0.018**	1.7 (−0.09–3.48)	0.063
Conventional model + arginine	24.43 (−3.80–52.66)	0.090	1.11 (−0.49–2.71)	0.173
Conventional model + urea	31.97 (3.87–60.08)	**0.026**	1.06 (−0.58–2.70)	0.204
Conventional model + GABR	33.80 (6.02–61.57)	**0.017**	3.95 (1.17–6.72)	**0.005**

MMD, moyamoya disease; NRI, net reclassification improvement; IDI, integrated discrimination improvement; OR, odds ratio; GABR, global arginine bioavailability ratio.

The conventional model included age, gender, heart rate, SBP, DBP, BMI, WBC count, lymphocyte count, platelet count, glucose, creatinine, uric acid, albumin, triglyceride, total cholesterol, HDL-C, LDL-C, apoA, apoB, and homocysteine. The bold values mean *p*-value < 0.05.

### Discussion

To our knowledge, this is the first clinical study to examine the prospective association between plasma urea cycle metabolites and the risk of adult MMD. In this study, we found that increased plasma ornithine levels were independently related to an increased risk of adult MMD. By contrast, increased arginine, urea, and GABR levels were associated with a decreased risk of adult MMD. These findings suggest that plasma urea cycle metabolites might be potential biomarkers for the risk of MMD.

The urea cycle is a metabolic pathway for the elimination of excess nitrogen and ammonia, which contain a range of amino acids and key enzymes. As a byproduct of the urea cycle, ornithine serves as a crucial building block for the production of proline, polyamines, and citrulline (Sivashanmugam et al., 2017). In this study, we discovered a positive correlation between plasma ornithine levels and the risk of MMD and its subcategories. Although the interaction between ornithine and MMD is unclear, numerous hypotheses can be put forth. First, the mitochondria in endothelial cells from MMD patients have aberrant morphology and function, according to the available information. This finding raises the possibility that MMD is a mitochondrial disease (Choi et al., 2018). Therefore, the activity of key enzymes in mitochondria related to the urea cycle (carbamoyl phosphate synthetase 1 and ornithine transcarbamylase) may be reduced in patients with MMD. Second, the urea cycle also occurs in enterocytes (Su et al., 2021). Numerous pro-inflammatory and anti-inflammatory cytokines affect the key enzymes of the urea cycle in non-hepatic cells (Morris 2002). Moreover, it has been demonstrated that systemic inflammation is critical in the pathophysiology of MMD (Han et al., 2020). Thus, it is reasonable to believe that elevated inflammatory factors in the circulation of patients with MMD may affect the activity of key enzymes of the urea cycle.

Arginine is an amino acid associated with many biological processes, including protein synthesis, immune reaction, the urea cycle, and the creation of nitric oxide (Morris, 2016). The correlation between plasma arginine levels and cardio-cerebrovascular diseases has been extensively studied. Many studies have indicated that higher levels of arginine in plasma were related to a lower incidence of cardio-cerebrovascular diseases (Ahmad et al., 2016; Yu et al., 2017; Grosse et al., 2020). In this study, we also discovered that plasma arginine levels are negatively related to the risk of MMD and its subcategories. Multiple potential pathophysiologic routes have been hypothesized for these inverse relationships. First, several studies have shown that arginine has anti-inflammatory effects. Qiu et al. found that arginine could inhibit the inflammatory response and oxidative stress induced by lipopolysaccharide (Qiu et al., 2019). In addition, a recent study suggested that arginine can reduce inflammation by preventing nuclear factor-κB activation (Liang et al., 2022). It has been demonstrated that the enhancement of nuclear factor-κB activation is involved in the pathogenesis of MMD (Takeda et al., 2020). Second, patients with mitochondrial disorders have nitric oxide deficiency (Almannai and El-Hattab, 2021). A case–control study indicated that nitric oxide deficiency may play a critical role in the onset of MMD (Park et al., 2014). Endothelial NO prevents platelet and leukocyte adhesion, attenuates inflammatory mediators, and causes endothelium-dependent vasorelaxation (Greco et al., 2018). As a result, it seems sensible to assume that arginine may prevent MMD through the production of nitric oxide. Third, another potential mechanism that appears to be present is the microbiome. In the case of Crohn’s disease, a higher prevalence of *Ruminococcus gnavus* and a decrease in *Roseburia inulinivorans* were observed, similar to what was observed in patients with MMD. Furthermore, there were elevated levels of microbial arginine and isoprene pathways in the gut microbiome of individuals with Crohn’s disease, indicating a possible association between the gut microbiome and uric cycle metabolism (Hu et al., 2021; Mineharu et al., 2022).

Although urea is the end product of the urea cycle, it has been associated with the development of stroke (You et al., 2018; Peng et al., 2021). Geng et al. showed in their metabolomic study that urea was lower in patients with MMD than in controls (Geng et al., 2020). In this study, we discovered that plasma urea levels were negatively related to the risk of MMD and its subcategories. As mentioned above, decreased activity of key enzymes in the urea cycle can lead to abnormal production of urea cycle metabolites, which also includes a decrease in the end product. A previous study suggested that the concept of GABR could be more predictive of the occurrence and development of cardio-cerebrovascular diseases. Our study also provides further evidence that GABR can also be a reliable predictor of MMD. Moreover, our results indicate that MMD patients with lower plasma levels of citrulline or urea and higher levels of GABR tend to have the RNF213 p.R4810K variant. Considering that GABR was inversely associated with MMD and was positively associated with the p.R4810K mutation, the relationship between the p.R4810K mutation and the urea cycle is still unclear. However, previous reports in this area are scarce and warrant further research.

Collateral vessels and the potential for angiogenesis differ among MMD subcategories (Zhao et al., 2019). However, the pathophysiological mechanisms of MMD subcategories remain unclear. A recent study showed that arginine can improve ventricular function by inducing neovascularization (Ranjbar, 2022). Our study demonstrated that increased GABR levels were associated with the risk of hemorrhagic MMD in overall MMD cases. In addition, MMD patients with higher GABR levels tended to have a higher Suzuki stage level. Therefore, GABR levels may be associated with the formation of collateral vessels in MMD. However, high GABR decreased the risk of MMD in the present study. We speculated that the possible reason for this contradictory conclusion is that the MMD cases we collected were at different Suzuki stages. Therefore, our next step was to perform a stratified analysis of MMD patients at different Suzuki stages. Another study has shown that homozygous or heterozygous RNF213 p.R4810K may be a potential biomarker for categorizing various clinical subcategories of MMD (Wang et al., 2021). The relationship between the p.R4810K mutation and the urea cycle remains to be explored further. One study showed that p.A4399T was associated with hemorrhagic MMD in a Chinese population (Wu et al., 2012). However, we did not detect the p.A4399T variant in the present study. We will continue to clarify the relationship between p.A4399T and GABR in MMD in future studies.

This study has several restrictions. First, this study was an observational study; the number of participants was not equal between the groups. Second, these findings could not be extended to children or other races, as only Chinese adult patients with MMD were included. Third, we only measured preoperative plasma urea cycle metabolite concentrations during hospitalization; this prevented us from studying the relationship between dynamic variations in plasma urea cycle metabolites and the prognosis of MMD. Finally, this is a prospective study using clinical information from a single institute. The applicability of the results is constrained, necessitating additional validation in a different cohort.

## Conclusion

In conclusion, increased plasma ornithine levels were independently related to an increased risk of adult MMD. By contrast, increased arginine, urea, and GABR levels were associated with a decreased risk of adult MMD. Adding arginine and GABR to conventional risk factors could improve risk prediction for MMD. These findings suggest that plasma urea cycle metabolites might be potential biomarkers for the risk of MMD.

## Data availability statement

The raw data supporting the conclusions of this article will be made available by the authors, without undue reservation.

## Ethics statement

The studies involving human participants were reviewed and approved by The Ethics Committee of Beijing Tiantan Hospital, Capital Medical University. The patients/participants provided their written informed consent to participate in this study.

## Author contributions

XiY: data curation (lead) and writing of the original draft (lead). PG and DZ: conceptualization (equal) and methodology (equal). YuZ and WL: visualization (equal) and investigation (equal). QZ, XuY, and JZ: supervision (equal). XL, RW, and YaZ: software (equal) and validation (equal). All authors contributed to the article and approved the submitted version.

## Funding

This study was supported by the National Key Technology Research and Development Programme of the Ministry of Science and Technology of China (2021YFC2500502).

## Conflict of interest

The authors declare that the research was conducted in the absence of any commercial or financial relationships that could be construed as a potential conflict of interest.

## Publisher’s note

All claims expressed in this article are solely those of the authors and do not necessarily represent those of their affiliated organizations, or those of the publisher, the editors and the reviewers. Any product that may be evaluated in this article, or claim that may be made by its manufacturer, is not guaranteed or endorsed by the publisher.

## Footnotes

^1^ https://www.r-project.org

## Glossary

MMD: moyamoya disease
SBP: systolic blood pressure
DBP: diastolic blood pressure
HR: heart rate
BMI: body mass index
WBC: white blood cells
LY: lymphocytes
PLT: platelets
Glu: glucose
ALB: albumin
Cr: creatinine
UA: uric acid
TG: triglyceride
TC: total cholesterol
HDL-C: high-density lipoprotein cholesterol
LDL-C: low-density lipoprotein cholesterol
apoA: apolipoprotein A
apoB: apolipoprotein B
Hcy: homocysteine
ORs: odd ratios
GABR: global arginine bioavailability ratio
DSA: digital subtraction angiography
