# Supplementary material for: Plasma urea cycle metabolite levels and the risk of moyamoya disease
Source: Front Neurosci. 2023 Jul 10;17:1163733. doi: 10.3389/fnins.2023.1163733 (PMC10363741; doi:10.3389/fnins.2023.1163733)
Supplement: Supplementary file 1 [file Data_Sheet_1.PDF]

Supplementary materials

Title: Plasma urea cycle metabolites and risk of moyamoya disease

Short title:

Figure I. Flow diagram of the study participants.

Table I. Baseline characteristics of participants according to quartiles of changes in ornithine

Table II. Baseline characteristics of participants according to quartiles of changes in arginine

Table III. Baseline characteristics of participants according to quartiles of changes in urea

Table IV. Baseline characteristics of participants according to quartiles of changes in GABR

Table V. Comparison of plasma urea cycle metabolites between different Suzuki stage or RNF213 p.R4810K groups in MMD patients

Table VI. The association between GABR and the risk of hemorrhagic MMD in the overall MMD cases

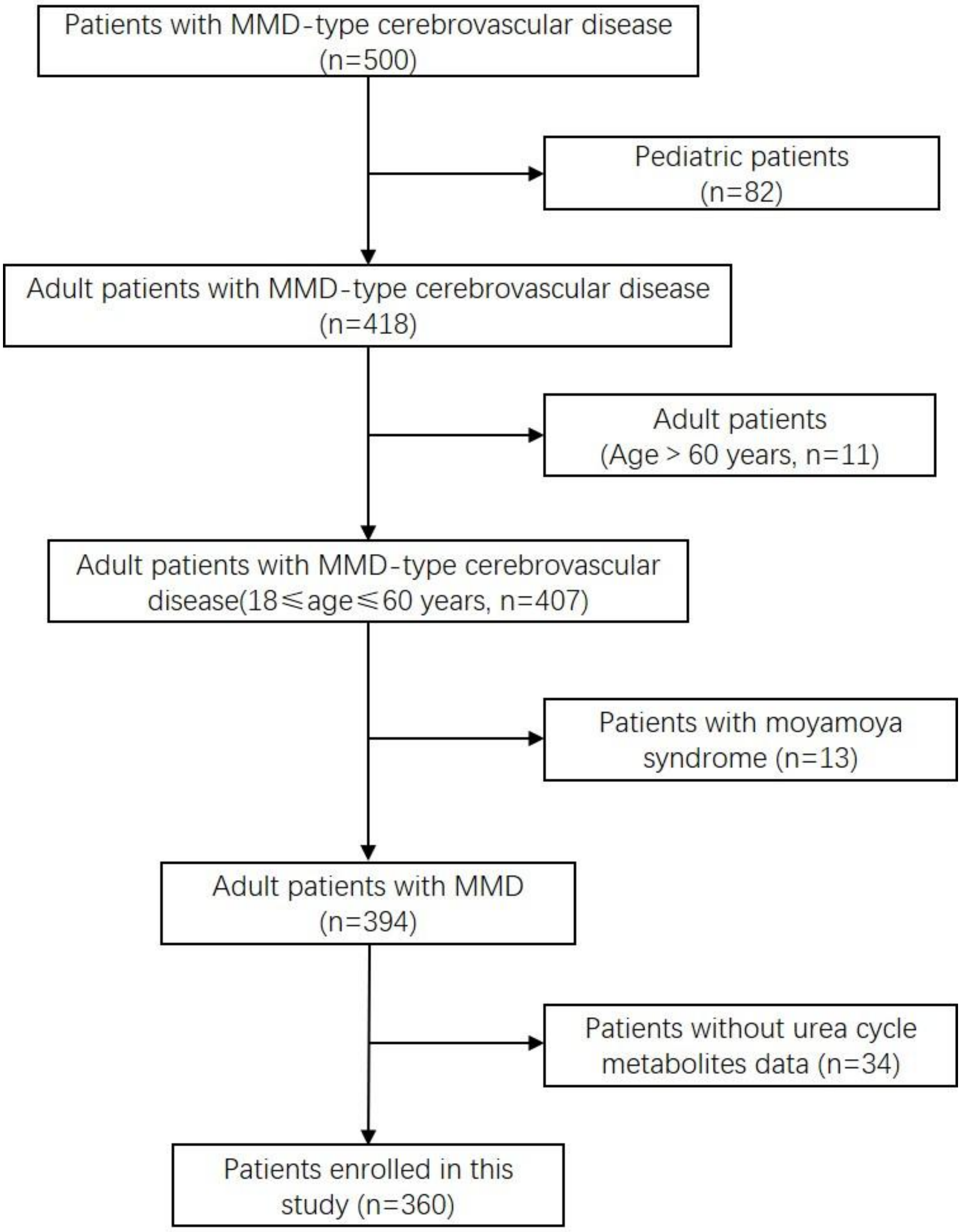

Figure I. Flow diagram of the study participants.

MMD indicates moyamoya disease;

Table I. Baseline characteristics of participants according to quartiles of changes in ornithine

Variables	Quartiles of changes in ornithine				***P* for trend**
	Q1 (<28.61)	Q2 (28.61-<35.61)	Q3 (35.61-<42.84)	Q4 (≥42.84)	
No. of participants	112	113	112	112	
Age, years	35.50 (28.00-45.00)	45.00 (34.50-49.50)	43.50 (33.25-50.00)	46.00 (38.00-52.00)	**0.000**
Men (%)	17 (15.18)	39 (34.51)	57 (50.89)	74 (66.07)	**0.000**
Medical history (%)					
Hypertension	22 (19.64)	33 (29.20)	33 (29.46)	43 (38.39)	**0.003**
Diabetes	7 (6.25)	16 (14.16)	15 (13.39)	21 (18.75)	**0.010**
Coronary artery disease	0 (0)	2 (1.77)	0 (0)	5 (4.46)	**0.027**
Hyperlipidemia	11 (9.82)	10 (8.85)	17 (15.18)	16 (14.29)	0.151
Thyroid disease	6 (5.36)	4 (3.54)	4 (3.57)	7 (6.25)	0.761
Smoking	5 (4.46)	15 (16.67)	23 (20.54)	28 (25.00)	**0.000**
Drinking	1 (0.89)	10 (8.85)	12 (10.71)	19 (16.96)	**0.000**
Clinical feature					
Heart rate, bpm	78.00 (72.00-80.00)	78.00 (75.50-80.00)	78.00 (74.25-80.00)	78.00 (74.00-80.00)	0.754
SBP, mmHg	126.00 (120.00-137.00)	132.00 (122.00-140.00)	130.00 (120.25-140.00)	132.00 (127.00-140.00)	**0.001**
DBP, mmHg	79.50 (74.00-84.75)	81.00 (76.50-90.00)	79.00 (75.00-86.75)	83.50 (77.00-89.00)	**0.026**
BMI, kg/m^2	23.58 (21.34-26.14)	24.22 (22.32-26.50)	25.52 (22.77-27.95)	25.76 (23.03-29.36)	**0.000**
Laboratory results, median (IQR)					
WBC count, $10^9/L$	6.48 (5.38-7.68)	6.60 (5.58-7.68)	6.74 (5.48-7.70)	6.88 (5.72-8.37)	0.141
Lymphocyte count, $10^9/L$	1.90 (1.51-2.28)	1.89 (1.51-2.39)	1.99 (1.62-2.41)	1.92 (1.47-2.34)	0.411
Platelet count, $10^9/L$	261.50	245.00 (206.00-303.50)	243.50	236.50	**0.018**

	(220.25-294.75)		(198.50-291.25)	(200.25-266.50)	
Glucose, mmol/L	4.92 (4.60-5.38)	5.13 (4.74-5.65)	5.05 (4.75-5.49)	5.19 (4.81-5.90)	**0.042**
Creatinine, µmol/L	48.50 (43.23-55.60)	55.10 (46.55-65.75)	58.95 (49.83-68.75)	62.70 (51.23-71.20)	**0.000**
Uric acid, µmol/L	277.45 (224.88-324.83)	304.20 (238.50-359.40)	319.65 (271.65-381.70)	339.35 (275.40-411.95)	**0.000**
Albumin, g/L	45.25 (43.53-46.88)	45.10 (42.90-47.00)	45.65 (43.50-47.50)	45.30 (43.50-47.60)	0.150
Triglyceride, mmol/L	1.00 (0.71-1.48)	1.06 (0.75-1.68)	1.25 (0.83-1.58)	1.22 (0.83-1.68)	0.110
Total cholesterol, mmol/L	4.24 (3.70-4.82)	4.26 (3.64-4.93)	4.23 (3.59-4.76)	4.36 (3.72-5.00)	0.854
HDL-C, mmol/L	1.44 (1.20-1.67)	1.34 (1.15-1.53)	1.32 (1.14-1.47)	1.28 (1.10-1.47)	**0.000**
LDL-C, mmol/L	2.40 (1.96-2.96)	2.45 (1.91-3.05)	2.40 (1.82-2.86)	2.49 (2.02-3.22)	0.641
apoA, g/L	1.36 (1.22-1.54)	1.31 (1.19-1.46)	1.27 (1.13-1.44)	1.30 (1.11-1.45)	**0.001**
apoB, g/L	0.77 (0.66-0.94)	0.81 (0.70-0.97)	0.82 (0.69-0.96)	0.86 (0.72-1.03)	**0.049**
Homocysteine, µmol/L	10.18 (7.97-13.18)	10.62 (8.92-13.51)	12.78 (9.62-16.45)	12.36 (10.33-15.63)	**0.000**
Citrulline, ng/mL	15.86 (13.56-19.17)	20.31 (16.77-22.94)	21.91 (18.64-25.50)	25.07 (21.66-28.86)	**0.000**
Arginine, ng/mL	63.34 (54.09-74.94)	67.19 (57.82-78.47)	65.79 (58.08-77.89)	70.68 (58.48-79.80)	**0.023**
Aspartic acid, ng/mL	50.13 (41.04-60.79)	56.07 (48.77-70.27)	57.83 (47.76-66.78)	61.65 (50.39-76.93)	**0.000**
Urea, mmol/L	4.94 (4.15-5.79)	5.74 (4.89-6.47)	5.94 (5.23-6.68)	6.37 (5.28-7.42)	**0.000**
GABR, median (IQR)	1.58 (1.39-1.95)	1.30 (1.11-1.52)	1.08 (0.96-1.24)	0.93 (0.78-1.08)	**0.000**

SBP indicates systolic blood pressure; DBP, diastolic blood pressure; BMI, body mass index; WBC, white blood cells; HDL-C, high-density lipoprotein cholesterol; LDL-C, low-density lipoprotein cholesterol; apoA, apolipoprotein A; apoB, apolipoprotein B; GABR, global arginine bioavailability ratio.

Table II. Baseline characteristics of participants according to quartiles of changes in arginine

Variables	Quartiles of changes in arginine				*P* for trend
	Q1 (<57.38)	Q2 (57.38-<66.41)	Q3 (66.41-<78.41)	Q4 (≥78.41)	
No. of participants	112	112	113	112	
Age, years	42.50 (31.00-50.00)	39.50 (33.00-47.00)	43.00 (33.00-52.00)	44.00 (34.00-51.75)	0.059
Men (%)	43 (38.39)	39 (34.82)	49 (43.36)	56 (50.00)	**0.037**
Medical history (%)					
Hypertension	29 (25.89)	35 (31.25)	33 (29.20)	34 (30.36)	0.555
Diabetes	17 (15.18)	11 (9.82)	11 (9.73)	20 (17.86)	0.579
Coronary artery disease	1 (0.89)	1 (0.89)	4 (3.54)	1 (0.89)	0.611
Hyperlipidemia	14 (12.50)	10 (8.93)	16 (14.16)	14 (12.50)	0.703
Thyroid disease	4 (3.57)	5 (4.46)	5 (4.42)	7 (6.25)	0.370
Smoking	16 (14.29)	14 (12.50)	20 (17.70)	21 (18.75)	0.228
Drinking	7 (6.25)	10 (8.93)	13 (11.50)	12 (10.71)	0.194
Clinical feature					
Heart rate, bpm	78.00 (74.00-80.00)	78.00 (74.00-80.00)	78.00 (72.50-80.00)	78.00 (75.00-85.00)	0.170
SBP, mmHg	130.00 (120.25-138.00)	131.50 (122.00-140.00)	130.00 (124.00-139.50)	130.00 (122.25-140.00)	0.600
DBP, mmHg	80.00 (75.00-86.75)	82.00 (77.00-89.75)	80.00 (76.00-87.00)	80.00 (74.00-88.75)	0.555
BMI, kg/m^2	24.15 (21.61-26.80)	24.64 (22.29-27.84)	24.44 (22.26-27.39)	25.97 (23.42-28.04)	**0.004**
Laboratory results, median (IQR)					
WBC count, $10^9/L$	6.51 (5.58-7.57)	6.64 (5.51-8.18)	6.47 (5.28-7.90)	6.88 (6.03-8.14)	0.544
Lymphocyte count, $10^9/L$	1.88 (1.45-2.29)	1.99 (1.54-2.42)	1.83 (1.54-2.26)	2.01 (1.61-2.55)	0.224
Platelet count, $10^9/L$	236.00	248.00 (202.00-288.50)	245.00	251.50	**0.000**

	(202.25-268.50)		(209.00-281.00)	(216.25-318.50)	
Glucose, mmol/L	5.12 (4.63-5.82)	4.96 (4.63-5.54)	5.11 (4.75-5.66)	5.16 (4.79-5.62)	0.895
Creatinine, µmol/L	54.05 (46.25-66.25)	53.80 (46.23-65.00)	57.20 (47.70-68.05)	58.00 (48.88-67.40)	0.081
Uric acid, µmol/L	304.70 (254.33-361.65)	282.90 (235.00-348.95)	320.40 (256.85-368.95)	320.95 (271.85-398.35)	0.007
Albumin, g/L	45.70 (43.60-47.08)	45.70 (43.63-47.30)	44.90 (43.30-47.40)	45.05 (43.20-46.85)	0.416
Triglyceride, mmol/L	1.22 (0.82-1.72)	1.07 (0.71-1.47)	1.17 (0.81-1.53)	1.08 (0.75-1.62)	0.593
Total cholesterol, mmol/L	4.23 (3.41-4.76)	4.30 (3.69-4.95)	4.23 (3.69-4.79)	4.46 (3.82-5.00)	0.076
HDL-C, mmol/L	1.30 (1.15-1.51)	1.34 (1.14-1.58)	1.34 (1.15-1.57)	1.33 (1.15-1.53)	0.909
LDL-C, mmol/L	2.31 (1.79-3.03)	2.46 (1.86-3.03)	2.40 (1.90-2.93)	2.63 (2.10-3.20)	**0.024**
apoA, g/L	1.30 (1.13-1.47)	1.31 (1.18-1.49)	1.32 (1.19-1.52)	1.30 (1.14-1.43)	0.999
apoB, g/L	0.79 (0.69-0.97)	0.82 (0.68-0.98)	0.78 (0.69-0.95)	0.87 (0.70-0.99)	0.266
Homocysteine, µmol/L	12.31 (9.00-14.20)	10.86 (8.74-13.40)	11.42 (9.22-13.88)	11.50 (9.43-15.53)	0.146
Ornithine, ng/mL	33.93 (27.17-41.14)	35.24 (28.70-41.07)	36.13 (29.85-43.36)	36.46 (29.01-45.71)	**0.020**
Citrulline, ng/mL	19.29 (15.11-22.13)	19.97 (16.44-23.43)	21.76 (17.99-25.98)	23.22 (18.77-27.20)	**0.000**
Aspartic acid, ng/mL	54.27 (46.48-64.78)	56.31 (45.53-66.89)	55.24 (45.14-69.79)	58.82 (49.76-76.95)	**0.002**
Urea, mmol/L	5.58 (4.92-6.51)	5.55 (4.62-6.34)	5.80 (4.91-6.67)	5.98 (4.84-7.10)	0.084
GABR, median (IQR)	0.96 (0.78-1.25)	1.13 (0.96-1.33)	1.26 (1.05-1.47)	1.53 (1.23-1.83)	**0.000**

SBP indicates systolic blood pressure; DBP, diastolic blood pressure; BMI, body mass index; WBC, white blood cells; HDL-C, high-density lipoprotein cholesterol; LDL-C, low-density lipoprotein cholesterol; apoA, apolipoprotein A; apoB, apolipoprotein B; GABR, global arginine bioavailability ratio.

Table III. Baseline characteristics of participants according to quartiles of changes in urea

Variables	Quartiles of changes in urea				*P* for trend
	Q1 (<4.87)	Q2 (4.87-<5.69)	Q3 (5.69-<6.59)	Q4 (≥6.59)	
No. of participants	112	112	113	112	
Age, years	39.00 (31.25-47.00)	41.50 (33.00-50.00)	44.00 (33.00-50.00)	45.00 (35.00-52.00)	**0.001**
Men (%)	33 (29.46)	47 (41.96)	49 (43.36)	58 (51.79)	**0.001**
Medical history (%)					
Hypertension	30 (26.79)	33 (29.46)	28 (24.78)	40 (35.71)	0.251
Diabetes	14 (12.50)	12 (10.71)	17 (15.04)	16 (14.29)	0.497
Coronary artery disease	0 (0)	0 (0)	4 (3.54)	3 (2.68)	**0.027**
Hyperlipidemia	11 (9.82)	13 (11.61)	17 (15.04)	13 (11.61)	0.521
Thyroid disease	7 (6.25)	4 (3.57)	6 (5.31)	4 (3.57)	0.481
Smoking	16 (14.29)	16 (14.29)	23 (20.35)	16 (14.29)	0.692
Drinking	5 (4.46)	12 (10.71)	15 (13.27)	10 (8.93)	0.194
Clinical feature					
Heart rate, bpm	78.00 (75.00-80.00)	78.00 (73.25-80.00)	78.00 (74.00-80.00)	78.00 (73.25-80.75)	0.777
SBP, mmHg	130.00 (120.00-140.00)	131.50 (124.00-140.00)	130.00 (122.00-139.50)	130.00 (120.00-138.00)	0.359
DBP, mmHg	80.00 (75.00-87.75)	80.50 (75.00-89.00)	80.00 (76.50-86.50)	81.00 (75.00-88.75)	0.768
BMI, kg/m^2	24.63 (21.78-26.91)	25.23 (22.73-28.07)	24.73 (22.61-27.24)	24.84 (22.38-27.68)	0.711
Laboratory results, median (IQR)					
WBC count, 10^9/L	7.12 (5.73-8.17)	6.57 (5.39-8.08)	6.47 (5.50-7.95)	6.54 (5.56-7.34)	0.267
Lymphocyte count, 10^9/L	2.00 (1.55-2.42)	1.99 (1.54-2.45)	1.89 (1.47-2.25)	1.87 (1.56-2.32)	0.358
Platelet count, 10^9/L	263.00	243.50 (204.00-269.75)	245.00	234.00	0.072

	(216.00-309.25)		(204.50-292.00)	(204.25-270.00)	
Glucose, mmol/L	5.02 (4.65-5.59)	5.15 (4.82-5.72)	5.00 (4.62-5.39)	5.15 (4.88-5.85)	0.474
Creatinine, µmol/L	50.35 (44.30-62.95)	54.45 (46.23-65.98)	57.30 (47.00-67.60)	62.85 (51.58-70.70)	**0.000**
Uric acid, µmol/L	298.80 (236.95-360.68)	302.60 (247.13-357.60)	309.90 (252.05-367.75)	325.55 (271.18-386.70)	**0.002**
Albumin, g/L	45.70 (43.73-47.38)	45.20 (42.90-47.00)	44.60 (43.00-46.85)	45.70 (43.83-47.48)	0.698
Triglyceride, mmol/L	1.01 (0.74-1.47)	1.21 (0.79-1.57)	1.15 (0.80-1.53)	1.18 (0.76-1.70)	0.962
Total cholesterol, mmol/L	4.23 (3.53-4.88)	4.13 (3.56-4.72)	4.28 (3.69-4.88)	4.46 (3.88-5.06)	0.207
HDL-C, mmol/L	1.34 (1.15-1.58)	1.33 (1.12-1.51)	1.32 (1.15-1.54)	1.34 (1.15-1.53)	0.642
LDL-C, mmol/L	2.40 (1.90-2.98)	2.24 (1.78-2.88)	2.45 (2.03-3.03)	2.61 (2.13-3.19)	0.090
apoA, g/L	1.33 (1.19-1.49)	1.30 (1.15-1.50)	1.30 (1.15-1.43)	1.30 (1.16-1.48)	0.128
apoB, g/L	0.78 (0.67-0.99)	0.80 (0.65-0.95)	0.84 (0.71-0.97)	0.82 (0.72-0.98)	0.470
Homocysteine, µmol/L	11.80 (9.13-14.58)	11.36 (9.22-15.56)	10.50 (8.90-13.06)	12.01 (9.73-14.00)	0.899
Ornithine, ng/mL	30.75±9.86	36.25±11.93	36.85±10.67	41.07±10.82	**0.000**
Citrulline, ng/mL	17.38±5.43	20.31±4.82	21.58±5.76	24.94±5.97	**0.000**
Arginine, ng/mL	68.84±16.80	66.24±15.73	69.64±18.08	71.00±17.03	0.168
Aspartic acid, ng/mL	57.31±19.32	56.83±14.17	57.82±18.94	64.28±18.14	**0.004**
GABR, median (IQR)	1.39 (1.16-1.81)	1.19 (0.94-1.45)	1.19 (0.99-1.43)	1.07 (0.88-1.33)	**0.000**

SBP indicates systolic blood pressure; DBP, diastolic blood pressure; BMI, body mass index; WBC, white blood cells; HDL-C, high-density lipoprotein cholesterol; LDL-C, low-density lipoprotein cholesterol; apoA, apolipoprotein A; apoB, apolipoprotein B; GABR, global arginine bioavailability ratio.

Table IV. Baseline characteristics of participants according to quartiles of changes in GABR

Variables	Quartiles of changes in GABR				*P* for trend
	Q1 (<0.98)	Q2 (0.98-<1.20)	Q3 (1.20-<1.48)	Q4 (≥1.48)	
No. of participants	113	112	112	112	
Age, years	46.00 (35.00-52.00)	46.00 (35.25-50.00)	40.00 (32.00-48.75)	37.50 (31.00-47.00)	**0.000**
Men (%)	65 (57.52)	48 (42.86)	40 (35.71)	34 (30.36)	**0.000**
Medical history (%)					
Hypertension	36 (31.86)	37 (33.04)	33 (29.46)	25 (22.32)	0.094
Diabetes	21 (18.58)	14 (12.50)	12 (10.71)	12 (10.71)	0.074
Coronary artery disease	3 (2.65)	3 (2.68)	1 (0.89)	0 (0)	0.062
Hyperlipidemia	19 (16.81)	8 (7.14)	13 (11.61)	14 (12.50)	0.532
Thyroid disease	3 (2.65)	9 (8.04)	6 (5.36)	3 (2.68)	0.775
Smoking	24 (21.24)	22 (19.64)	13 (11.61)	12 (10.71)	**0.010**
Drinking	14 (12.39)	14 (12.50)	5 (4.46)	9 (8.04)	0.086
Clinical feature					
Heart rate, bpm	78.00 (72.50-80.00)	78.00 (75.00-80.00)	78.00 (74.00-80.00)	78.00 (75.00-80.00)	0.091
SBP, mmHg	132.00 (123.50-140.00)	130.00 (124.00-140.00)	130.50 (122.25-138.00)	127.00 (120.00-140.00)	**0.027**
DBP, mmHg	80.00 (75.00-88.50)	80.50 (75.00-89.00)	80.00 (76.00-86.75)	80.00 (75.00-87.75)	0.379
BMI, kg/m^2	25.00 (22.49-27.97)	25.04 (22.57-28.12)	24.57 (22.37-26.54)	24.71 (22.10-27.60)	0.199
Laboratory results, median (IQR)					
WBC count, 10^9/L	6.34 (5.44-7.67)	6.78 (5.46-8.23)	6.84 (5.64-7.75)	6.60 (5.78-7.82)	0.352
Lymphocyte count, 10^9/L	1.93 (1.54-2.38)	1.81 (1.46-2.22)	1.90 (1.53-2.35)	2.04 (1.62-2.52)	0.351
Platelet count, 10^9/L	232.00	247.50 (205.25-280.50)	246.00	264.00	**0.000**

	(189.00-257.50)		(208.00-295.00)	(217.50-304.50)	
Glucose, mmol/L	5.14 (4.77-5.88)	5.10 (4.73-5.62)	5.00 (4.61-5.48)	5.13 (4.77-5.60)	0.389
Creatinine, µmol/L	61.00 (50.50-71.25)	56.95 (46.63-67.75)	54.90 (47.00-65.28)	50.30 (45.03-63.50)	**0.000**
Uric acid, µmol/L	331.30 (278.00-395.15)	303.70 (253.98-361.93)	292.80 (238.90-371.90)	296.15 (237.68-352.55)	**0.000**
Albumin, g/L	45.70 (44.10-47.65)	45.00 (43.08-46.78)	44.95 (43.10-47.28)	45.35 (43.53-46.90)	0.223
Triglyceride, mmol/L	1.37 (0.95-1.82)	1.08 (0.74-1.37)	1.10 (0.79-1.53)	0.99 (0.71-1.67)	0.293
Total cholesterol, mmol/L	4.30 (3.57-4.99)	4.23 (3.50-4.76)	4.31 (3.69-4.96)	4.33 (3.84-4.89)	0.297
HDL-C, mmol/L	1.29 (1.13-1.49)	1.34 (1.15-1.52)	1.32 (1.15-1.58)	1.36 (1.15-1.57)	**0.044**
LDL-C, mmol/L	2.41 (1.83-3.09)	2.39 (1.83-2.89)	2.43 (1.97-3.21)	2.49 (1.99-2.98)	0.337
apoA, g/L	1.30 (1.13-1.46)	1.30 (1.17-1.50)	1.30 (1.15-1.44)	1.33 (1.19-1.50)	0.210
apoB, g/L	0.84 (0.70-1.01)	0.80 (0.68-0.92)	0.81 (0.69-1.04)	0.80 (0.69-0.94)	0.540
Homocysteine, µmol/L	12.40 (10.05-15.64)	11.85 (9.15-13.98)	10.98 (8.74-13.30)	10.75 (8.92-14.58)	**0.030**
Ornithine, ng/mL	44.40 (39.12-50.46)	38.85 (34.12-43.46)	31.30 (27.24-37.25)	25.87 (21.12-31.62)	**0.000**
Citrulline, ng/mL	23.60 (20.62-27.00)	21.75 (18.63-25.57)	19.68 (16.39-23.01)	16.10 (13.46-21.13)	**0.000**
Arginine, ng/mL	56.20 (49.66-64.12)	65.70 (59.10-74.52)	68.76 (59.35-78.55)	79.78 (66.90-91.69)	**0.000**
Aspartic acid, ng/mL	56.38 (48.67-71.25)	53.98 (47.60-64.00)	56.26 (47.09-70.28)	55.75 (44.63-69.57)	0.846
Urea, mmol/L	6.06 (5.37-7.10)	5.90 (5.17-6.65)	5.66 (4.85-6.37)	5.09 (4.20-6.04)	**0.000**

SBP indicates systolic blood pressure; DBP, diastolic blood pressure; BMI, body mass index; WBC, white blood cells; HDL-C, high-density lipoprotein cholesterol; LDL-C, low-density lipoprotein cholesterol; apoA, apolipoprotein A; apoB, apolipoprotein B; GABR, global arginine bioavailability ratio.

Table V. Comparison of plasma urea cycle metabolites between different Suzuki stage or RNF213 p.R4810K groups in MMD patients

Variables	Suzuki stage		*P* Value	RNF213 p.R4810K		*P* Value
	0-2	3-6		No	Yes	
Ornithine, ng/mL	36.82 (30.92-44.33)	35.69 (28.75-43.26)	0.211	36.61 (30.05-44.35)	34.59 (27.20-42.82)	0.061
Citrulline, ng/mL	20.31 (16.48-25.35)	21.05 (17.04-24.63)	0.867	21.07 (17.51-25.19)	18.96 (15.53-23.17)	**0.019**
Arginine, ng/mL	63.95 (55.09-73.47)	65.78 (57.37-77.16)	0.317	65.60 (57.39-75.91)	64.46 (55.29-74.51)	0.477
Aspartic acid, ng/mL	58.74 (49.98-70.91)	54.81 (45.46-65.28)	**0.049**	55.14 (47.49-66.91)	58.25 (45.95-70.54)	0.559
Urea, mmol/L	5.55 (4.81-6.40)	5.61 (4.84-6.47)	0.590	5.61 (4.75-6.53)	5.32 (4.53-5.96)	**0.036**
GABR	1.09 (0.88-1.35)	1.19 (1.00-1.46)	**0.035**	1.15 (0.95-1.41)	1.28 (1.00-1.53)	**0.046**

GABR indicates global arginine bioavailability ratio.

Table VI. The association between GABR and the risk of hemorrhagic MMD in the overall MMD cases.

GABR	No. of events (%)	Crude		Model 1*		Model 2†	
		OR (95% CI)	*P* Value	OR (95% CI)	*P* Value	OR (95% CI)	*P* Value
**Continuous**	101 (28.06)	1.317 (0.761-2.280)	0.324	1.239 (0.688-2.233)	0.475	1.410 (0.719-2.764)	0.317
**Natural log transformed**	101 (28.06)	1.670 (0.787-3.544)	0.181	1.536 (0.684-3.448)	0.299	1.870 (0.745-4.694)	0.183
**Quartiles**							
Q1 (<0.98)	22 (21.78)	1.0 (Ref)		1.0 (Ref)		1.0 (Ref)	
Q2 (0.98-<1.20)	26 (25.74)	1.302 (0.676-2.505)	0.430	1.297 (0.658-2.553)	0.453	1.240 (0.585-2.626)	0.575
Q3 (1.20-<1.48)	25 (24.75)	1.329 (0.686-2.575)	0.400	1.251 (0.626-2.498)	0.526	1.236 (0.581-2.629)	0.583
Q4 (≥1.48)	28 (27.72)	1.974 (1.016-3.834)	**0.045**	1.837 (0.911-3.705)	0.089	2.432 (1.088-5.436)	**0.030**

MMD indicates moyamoya disease; OR, odds ratio.

*Model 1 was adjusted for age, gender, heart rate, SBP, DBP, and BMI.

†Model 2 was adjusted for all the variables in model 1 plus WBC count, lymphocyte count, platelet count, glucose, creatinine, uric acid, albumin, triglyceride, total cholesterol, HDL-C, LDL-C, apoA, apoB, and homocysteine.
